# Toxicity profiles of cyclin-dependent kinase 4/6 inhibitors: safety analysis from clinical trials and the FDA adverse event reporting system

**DOI:** 10.3389/fonc.2025.1673284

**Published:** 2025-12-11

**Authors:** Jin Zhou, Xinmiao Lin, Shue Wang, Mumu Xie, Xiao Hu, Jiaqin Cai

**Affiliations:** 1 Department of Pharmacy, Fujian Maternity and Child Health Hospital, Fuzhou, China; 2 School of Pharmacy, Fujian Medical University, Fuzhou, China; 3 Information Management Department, Key Laboratory of Medical Big Data Engineering, Fuzhou University Affiliated Provincial Hospital, Fuzhou, China; 4 Department of Pharmacy, Fujian Provincial Hospital, Shengli Clinical Medical College of Fujian Medical University, Fuzhou University Affiliated Provincial Hospital, Fuzhou, China

**Keywords:** CDK4/6, inhibitors, toxicity, profiles, pharmacovigilance, network, meta, pharmacoepidemiology

## Abstract

**Aims:**

This study aimed to evaluate the comprehensive safety profile of CDK4/6 inhibitors in breast cancer treatment by analyzing real-world adverse event (AE) data and systematically reviewing clinical trial findings to provide evidence-based safety guidance.

**Methods:**

A disproportionality analysis based on real-world data from the FDA adverse event reporting system (FAERS) was combined with a systematic review to evaluate adverse events (AEs) associated with CDK4/6 inhibitors. A total of 16 studies involving 6,722 patients with advanced breast cancer were included to analyze the toxicity risks of CDK4/6 inhibitor combination therapies. Temporal patterns of AEs were assessed using FAERS data, and signal strengths were determined using the reporting odds ratio (ROR).

**Results:**

Combination therapy with CDK4/6 inhibitors significantly increased the risk of grade 3–5 AEs. FAERS analysis revealed that most AEs occurred within the first month of treatment, with the lowest incidence between the second and sixth months, followed by a significant rise thereafter. All CDK4/6 inhibitors were associated with myelosuppression, gastrointestinal toxicity, interstitial lung disease (ILD), and QT prolongation. Disproportionality analysis for “Torsade de pointes/QT prolongation” identified Ribociclib as the only agent with a strong positive signal (ROR 6.16, 95% CI 5.54–6.84). Additionally, unexpected AEs such as peripheral neuropathy, taste disorders, and epistaxis were detected.

**Conclusion:**

Clinicians should prioritize monitoring for myelosuppression, gastrointestinal toxicity, and QT prolongation (particularly with Ribociclib) during the first month of CDK4/6 inhibitor therapy. Long-term surveillance beyond 6 months is crucial to detect delayed-onset AEs.

## 1 Introduction

HR-positive HER2-negative breast cancer accounts for approximately 60% of all primary breast cancer cases, and approximately 20% of early estrogen receptor (ER)-positive patients may experience local or distal recurrence after treatment (1, 2). Recent studies have revealed the importance of cycle-independent kinase 4 and 6 (CDK4/6) inhibitors in endocrine-resistant breast cancer (3–5). Phase III clinical trials have shown that CDK4/6 inhibitors combined with ET almost demonstrated statistically and clinically significant progression-free survival (PFS) and/or overall survival (OS) in first-line/second-line treatment of patients with HR-positive/HER-2 negative (HR +/HER2−) metastatic breast cancer (MBC) (6–12). The currently approved CDK4/6 inhibitors include Palbociclib, Ribociclib, and Abemaciclib. These drugs have shown differences in terms of their efficacy and safety profiles. Adjuvant use of Abemaciclib plus standard ET significantly reduced the risk of IDF (13). Therefore, Abemaciclib was approved to be used with endocrine therapy for adjunctive therapy in early breast cancer with HR+/HER2, lymph node-positive, and high risk of recurrence with KI-67 20%.

A better understanding of the real-world safety profile of CDK4/6 inhibitors in patients with HR+ breast cancer will lead to better compliance and fewer interruptions and reflect on the desirable progression-free survival and overall survival. With the wide use of CDK4/6 inhibitors in clinical practice, adverse reaction assessment in the real world is lacking. In the present study, we conducted a comprehensive assessment to examine the toxicity spectrum of CDK4/6 inhibitors in patients with breast cancer by summarizing evidence from RCTs and performing pharmacovigilance analysis with the data obtained from the Food and Drug Administration Adverse Event Reporting System (FAERS) database (14).

## 2 Methods

### 2.1 Network meta-analysis

PubMed, Embase, and Cochrane Central Register of Controlled Trials databases were systematically searched for relevant articles from September 2021 until June 2024. **Supplementary Table S1** lists the detailed search strategies. Studies that met the following criteria were included: (I) Phase II and Phase III randomized controlled trials in patients with HR+/HER2 metastasis or advanced BC; (II) the treatment regimen must include at least one of the three CDK4/6 inhibitors (Abemaciclib, Palbociclib, Ribociclib); (III) detailed data on AE must be reported. We also searched the Clinical Trial website by the NCT number of each article for more detailed information. Two authors independently extracted data, including study identification, clinical trial identification, first author name, year of publication, study stage, sample size, median age, and specific treatment protocol. The proportion of selected cutaneous AEs and the 95% CI of each CDK4/6 inhibitor treatment regimen were evaluated.

Bayesian network meta-analysis is performed by Markov Chain Monte Carlo (MCMC) simulation using R software (V.4.3.2). The fixed- and random-effects models were used to estimate event rates and their corresponding 95% confidence intervals. Each model was run with 10,000 burn-in and 50,000 sampling iterations, using a thinning interval of 10. Convergence was assessed by Gelman–Rubin diagnostics (potential scale reduction factor <1.05) and visual inspection of trace plots. Between-study variance (τ²) was estimated to quantify heterogeneity, and node-splitting analysis was applied to assess network consistency. Non-informative (vague) priors were adopted for treatment effects and variance parameters following standard Bayesian practice. Model fit was evaluated using the deviance information criterion (DIC).

### 2.2 Pharmacovigilance study

#### 2.2.1 Data source

We have selected data from 28 quarters (Q1 2016–Q2 2024) in the FAERS database and imported it into the MYSQL database software for filtering. We performed fuzzy matching using drug names “PALBOCICLIB,” “IBRANCE,” “RIBOCICLIB,” “KISQALI,” “ABEMACICLIB,” and “VERZENIO” in MYSQL and selected the generic names “PALBOCICLIB,” “RIBOCICLIB,” and “ABEMACICLIB” and brand names “IBRANCE,” “KISQALI,” and “VERZENIO” as the primary suspected drugs in the reports. Exclusion criteria included duplicated reports and reports missing information on age, gender, or event date (14). Generic and brand names of CDK4/6 inhibitors used in this study include “Palbociclib,” “IBRANCE,” “Ribociclib,” “KISQALI,” “Abemaciclib,” and “VERZENIO”. Since the FAERS database is accessible to the public and patient records are anonymized and deidentified, ethical clearance and informed consent are not required for this study.

#### 2.2.2 Definition of adverse events

The adverse events recorded in the FAERS database are categorized using the Preferred Terms (PTs) of the Medical Dictionary for Regulatory Activities (MedDRA) terminology, which contains 27 system organ classes (SOCs). In addition, PT can be linked to more than one SOC due to the “multiaxiality” of MedDRA. Accordingly, we used MedDRA (version 22.1) to classify adverse events in each report to the corresponding SOC levels. Within each SOC, important safety signals, counts of each adverse event detected using PTs, were reported to describe the most common adverse events in each SOC for every drug.

Disproportionate analysis was performed to detect the three CDK4/6 inhibitors’ class safety profiles using all existing PTs. Key toxicities identified were characterized in terms of PT-level specific signs/symptoms for further disproportionality analysis among three CDK4/6 inhibitors.

#### 2.2.3 Data mining algorithm

We performed a disproportionality analysis using the reporting odds ratio (ROR) to assess whether there is a signal for a potentially increased risk of drug-associated AE among CDK4/6 inhibitors. Reporting odds ratios (RORs) were carried out, and safety signals were considered significant if the ROR was 2.0 and the 95% confidence interval values exceeded 1.0. All analyses were performed using R (version 4.3.2) and Microsoft Excel 2010.

## 3 Results

### 3.1 Network meta-analysis

#### 3.1.1 Characteristics of studies included in the pooled analysis

**Figure 1:**
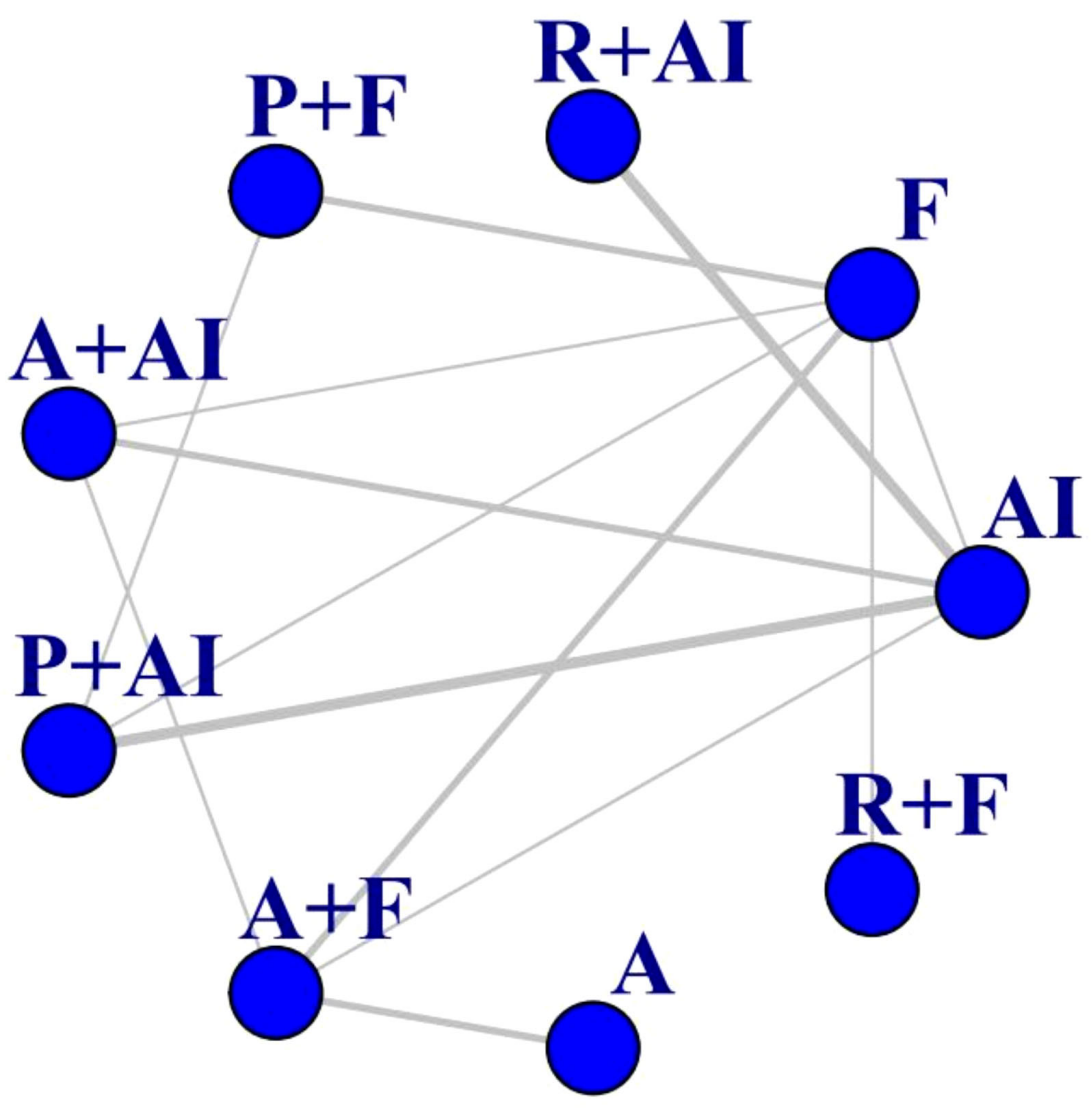
Network of comparisons for the Bayesian network meta-analysis. The thickness of the lines is proportional to the number of comparisons. AI, aromatase inhibitors; F, Fulvestrant; P, Palbociclib; A, Abemaciclib; R, Ribociclib.

A total of 16 studies with 6,722 patients with advanced breast cancer were included (9, 10, 15–28). As shown in the network diagram in **Figure 1**, this study analyzed a total of nine treatment regimens with aromatase inhibitors (1404), Fulvestrant (784), Palbociclib + AI (688), Palbociclib + Fulvestrant (829), Abemaciclib + AI (532), Abemaciclib + Fulvestrant (701), Abemaciclib (156), Ribociclib + AI (712), and Ribociclib + Fulvestrant (483). The detailed patient demographics and clinical characteristics are summarized in **Supplementary Table S2**.

#### 3.1.2 Network comparison for overall and grade 3–5 AEs

**Figure 2:**
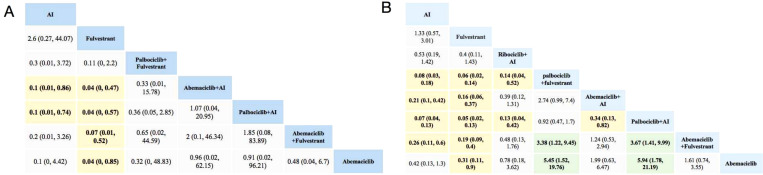
Toxicity profiles based on grade 1–5 adverse events **(A)** and grade 3–5 adverse events **(B)**. The estimated RR and its 95% confidence intervals between all treatments are shown in each cell. The column treatments are compared with the row treatment. RR > 1 (green squares) indicated that patients in the column treatment group had more AEs than patients in the row treatment group. RR < 1 (yellow squares) indicated that patients in the column treatment group had fewer AEs than patients in the row treatment group; significant results are in bold. For instance, as for AI in **Figure 2A**, patients receiving AI had a lower risk of grade 1–5 adverse events than those receiving Abemaciclib +AI (RR: 0.1, 95% CI: 0.01, 0.86). AI, aromatase inhibitor; AE, adverse event; RR, relative risk.

As shown in **Figure 2**, compared with using AI alone, Abemaciclib + AI and Palbociclib + AI are associated with a higher likelihood of experiencing adverse events of any grade. Treatment regimens involving Abemaciclib or Palbociclib in combination with AI or Fulvestrant are associated with higher rates of severe adverse events. In terms of serious adverse events, the ranking from safest to most risky is as follows: Fulvestrant, AI, Ribociclib+AI, Abemaciclib, Abemaciclib+Fulvestrant, Abemaciclib+AI, Palbociclib+Fulvestrant, and Palbociclib+AI. Palbociclib poses a higher risk of grade 3–5 adverse events compared with Abemacicli (OR = 5.85 [1.62, 21.08]) and Ribociclib (OR = 0.13 [0.04, 0.42]). Both Fulvestrant and AI demonstrate the lowest risk, whether in overall adverse events or specific system comparisons.

#### 3.1.3 Network comparison for specific AEs

**Figure 3:**
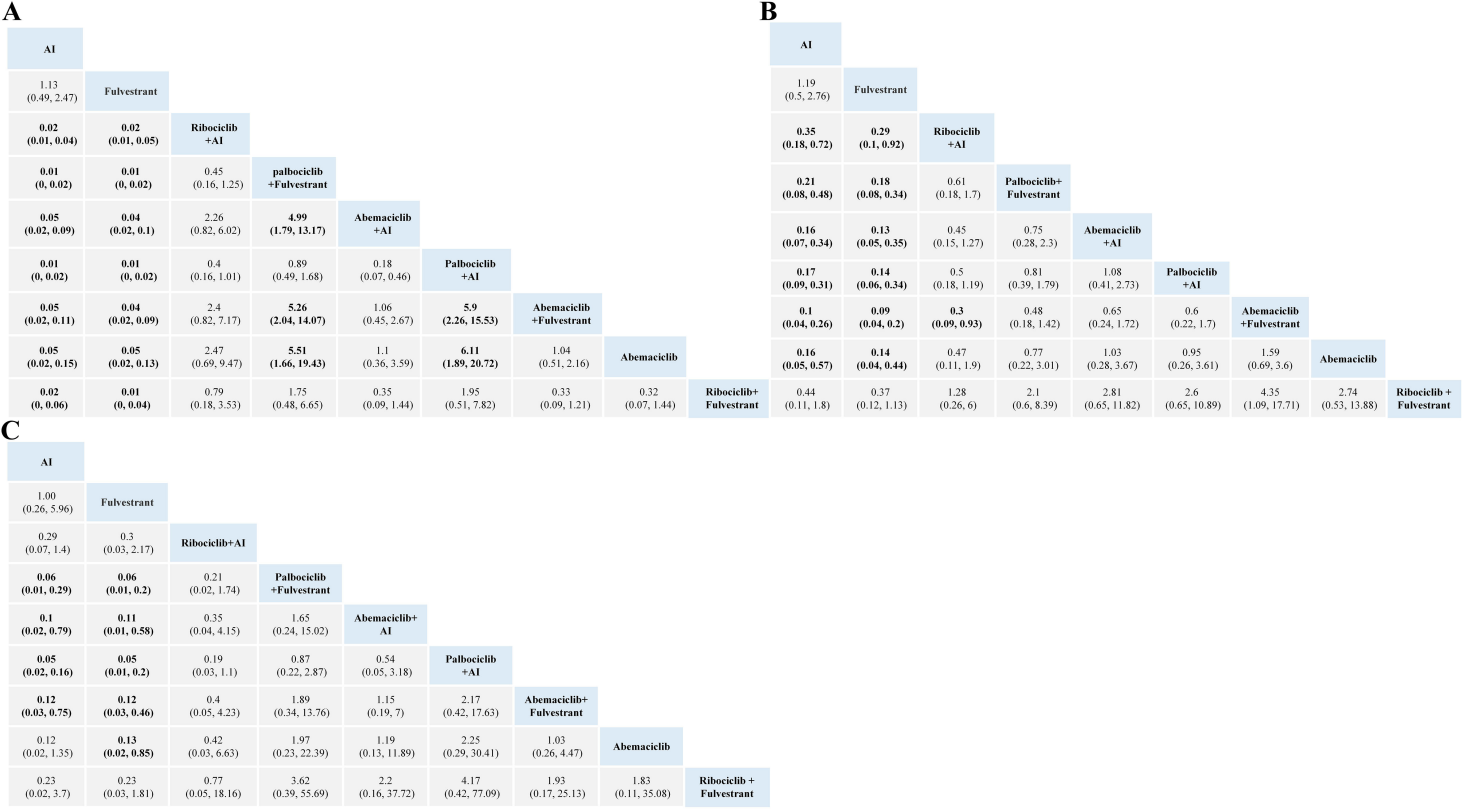
Toxicity profiles based on grade 1–5 neutropenia **(A)**, anemia **(B)** and thrombocytopenia **(C)**. The estimated RR and its 95% confidence intervals between all treatments are shown in each cell. The column treatments are compared with the row treatment. Significant results are in bold. AI, aromatase inhibitor; RR, relative risk.

For hematopoietic toxicity, as shown in **Figure 3**, compared with standalone endocrine therapy, the combination therapy of three CDK4/6 inhibitors with AI or Fulvestrant is more likely to result in neutropenia, thrombocytopenia and anemia. Neutropenia was more likely to occur with Palbociclib combination therapy than with Abemaciclib combination therapy (**Figure 3A**). For anemia and thrombocytopenia, the combination therapies of Abemaciclib and Palbociclib pose a higher risk than using aromatase inhibitors or Fulvestrant alone, with no statistical significance in comparison with Ribociclib (**Figures 3B, C**).

**Figure 4:**
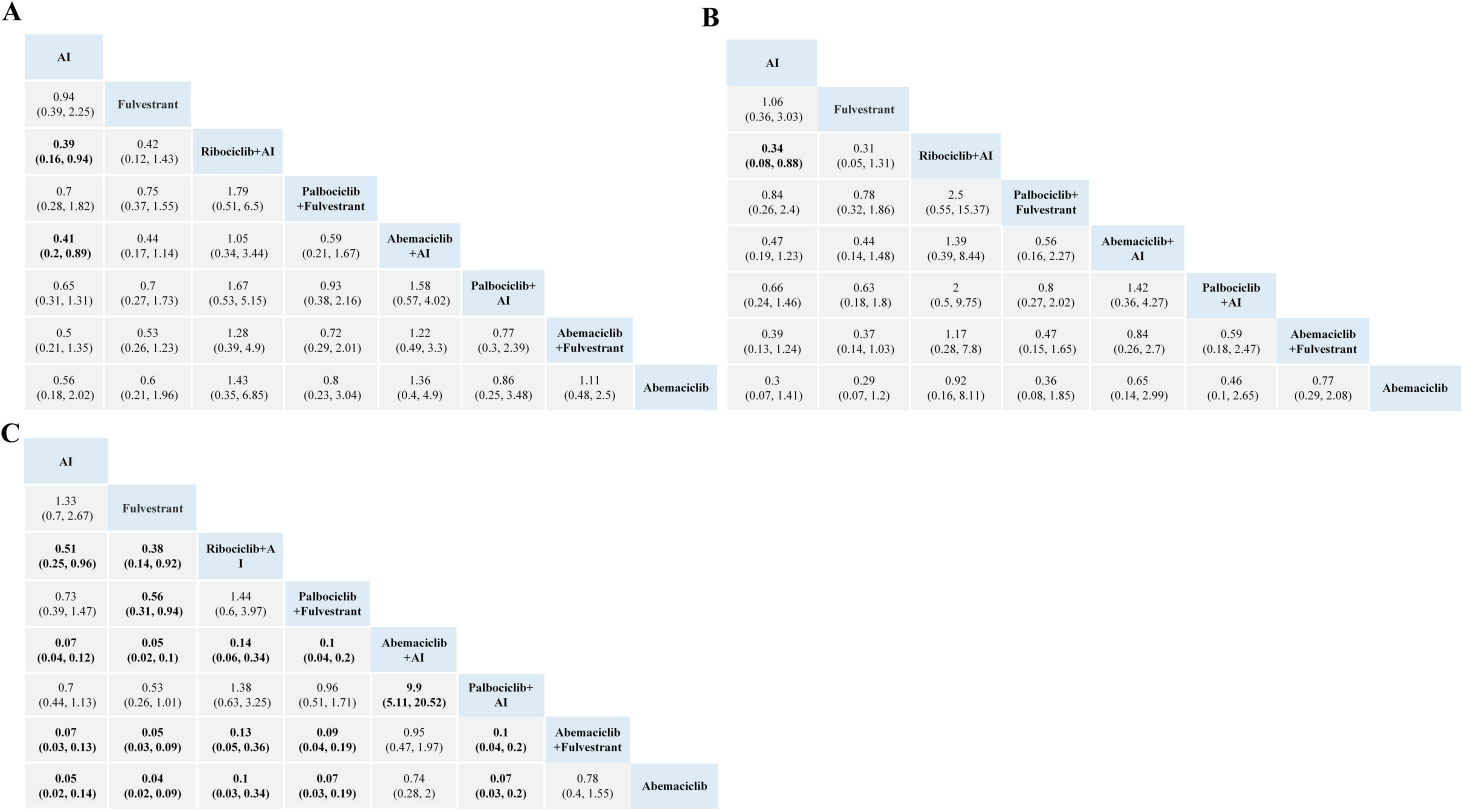
Toxicity profiles based on grade 1–5 nausea **(A)**, vomiting **(B)**, and diarrhea **(C)**. The estimated RR and its 95% confidence intervals between all treatments are shown in each cell. The column treatments are compared with the row treatment. Significant results are in bold.AI, aromatase inhibitor; RR, relative risk.

For gastrointestinal toxicity, Ribociclib + AI and Abemaciclib + AI are more likely to cause nausea than AI alone, as shown in **Figure 4**. The combinations of Abemaciclib are more likely to cause diarrhea. The safety ranking of the three CDK4/6 inhibitors in terms of diarrhea is as follows: Palbociclib, Ribociclib, Abemaciclib. Patients treated with Ribociclib + AI had the highest risk of nausea and vomiting (**Figures 4A, B**). Notably, patients treated with Abemaciclib monotherapy had the highest risk of diarrhea, even higher than those treated with various combination therapies (**Figure 4C**).

**Figure 5:**
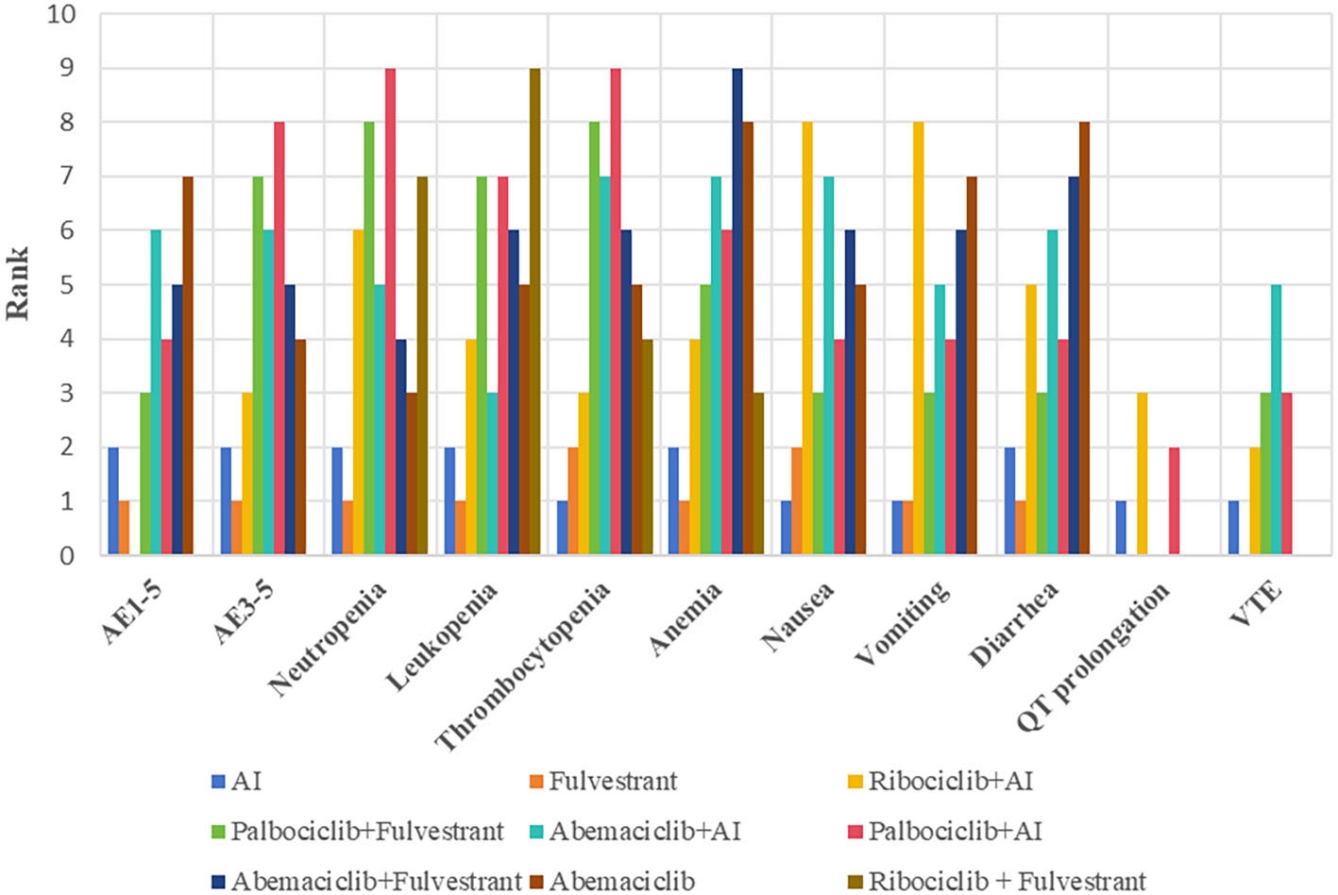
Evidence maps of adverse events based on nine treatments. The risk ranking was generated based on the surface under the cumulative ranking curves values, with a smaller value of ranking indicating a lower risk of adverse events. For instance, as for grade 1–5 adverse events, the safety of Fulvestrant ranked second among these treatments. AI, aromatase inhibitor; AE, adverse event; VTE, venous thromboembolism.

No significant difference in the risk of Q-T interval prolongation and thrombosis was observed between CDK4/6 inhibitors. Based on the network meta-analysis of existing data, the ranking of risk for QT prolongation is Ribociclib + AI, Palbociclib + AI, and AI. The risk levels for developing venous thromboembolism (VTE), from highest to lowest, are Abemaciclib, Palbociclib, Ribociclib, and Fulvestrant, shown in **Figure 5**.

### 3.2 Toxicity profiling of CDK4/6 inhibitors in disproportionality analysis

#### 3.2.1 Descriptive analysis

From January 1, 2011, to June 30, 2024, the FAERS database received a total of 92,427 AE reports for CDK4/6 inhibitors, with 70,227, 13,422, and 8,778 attributed to Palbociclib, Ribociclib, and Abemaciclib, respectively.

The majority of reports were from the USA. The average age and weight reported for CDK4/6 inhibitor users were different, and patients aged >45 years were the majority of AE reports. Most of the reports were submitted by consumers, who contributed to the highest percentage of reports for Palbociclib (42.96%), Ribociclib (50.13%), and Abemaciclib (43.08%). The characteristics of AE reports submitted for CDK4/6 inhibitors are described in **Table 1**.

**Table 1:** Characteristics of reports associated with CDK4/6 inhibitors from January 2011 to June 2024.

Characteristics	Palbociclib	Ribociclib	Abemaciclib
Number of events	70227	13422	8778
Age distribution, n (%)			
< 18y	63( 0.09)	19( 0.14)	4(0.05)
>18y, < 45y	3325(4.73)	862(6.42)	292(3.33)
≥45y, < 65y	23784(33.87)	2844(21.19)	1786(20.35)
≥65y, < 75y	18989(27.04)	1594(11.88)	1192(13.58)
>75y	14912(21.23)	974(7.26)	780(8.89)
Missing	9154(13.03)	7129(53.11)	4724(53.82)
Sex, n (%)			
Female	65761(93.64)	12244(91.22)	7808(88.95)
Male	1613(2.30)	244( 1.82)	152(1.73)
Missing	2853(4.06)	934(6.96)	818(9.32)
Report source, n (%)			
Physician	9394(13.38)	3391(25.26)	1180(13.44)
Pharmacist	20521(29.22)	1989(14.82)	2027(23.09)
Consumer	30408(43.30)	7038(52.44)	4262(48.55)
Other	9904(14.1)	1004(7.48)	1309(14.92)
Reporter country, n (%)			
USA	57558(81.96)	4639 (34.57)	6879(78.3)
Other countries	12669(18.04)	8783(65.43)	1899(21.7)

#### 3.2.2 Signal of system organ class and signal of preferred terms

The CDK4/6 inhibitor AE report signals based on SOCs are shown in **Table 2**, with 226,622, 59,437, and 19,672 attributed to Palbociclib, Ribociclib, and Abemaciclib, respectively. The most frequently reported SOCs using disproportionality analysis included “Gastrointestinal disorders,” “General disorders and administration site conditions,” “Neoplasms benign, malignant and unspecified (incl cysts and polyps),” and “Investigations,” both as a drug class and as a single agent. A list of the top 50 PTs associated with the most common statistically significant RORs for CDK4/6 inhibitors is shown in **Supplementary Table S3** 1-3. Our data mining revealed several significant AEs that were not explicitly mentioned in the three CDK4/6 inhibitors’ product labels. We found unexpected AEs consisting of PTs for Abemaciclib, such as abdominal pain, constipation, dysphagia, weight decreased, blood creatinine increased, dehydration, and blood potassium decreased. The unexpected AEs consist of PTs for Palbociclib, concluded constipation, dysphagia, pain, feeling abnormal, peripheral swelling, insomnia, oropharyngeal pain, arthralgia, back pain, pain in extremity, bone pain, and neuropathy. The unexpected AEs consisting of PTs for Ribociclib are peripheral swelling, decreased weight, dehydration, diabetes mellitus, pain in extremity, speech disorder, and taste disorder epistaxis. Our analysis has identified additional AEs that emphasize the overall comprehension of CDK4/6 inhibitors’ safety profile.

**Table 2:** Distribution of drug-reaction pairs attributed to CDK4/6 inhibitors according to relevant System Organ Class (SOCs)*.

SOC	Palbociclib		Ribociclib		Abemaciclib	
	n	%	n	%	n	%
General disorders and administration site conditions	40374	17.82	10504	17.67	3300	16.78
Investigations	30142	13.30	7137	12.01	1927	9.80
Gastrointestinal disorders	28886	12.75	6712	11.29	4892	24.87
Neoplasms benign, malignant and unspecified (incl cysts and polyps)	11722	5.17	4659	7.84	616	3.13
Skin and subcutaneous tissue disorders	14481	6.39	3691	6.21	859	4.37
Respiratory, thoracic and mediastinal disorders	11905	5.25	3371	5.67	1018	5.17
Nervous system disorders	14747	6.51	3369	5.67	939	4.77
Musculoskeletal and connective tissue disorders	11124	4.91	2960	4.98	395	2.01
Blood and lymphatic system disorders	9574	4.22	2514	4.23	809	4.11
Infections and infestations	9935	4.38	2363	3.98	755	3.84
Injury, poisoning and procedural complications	13571	5.99	2276	3.83	588	2.99
Psychiatric disorders	6322	2.79	1769	2.98	297	1.51
Metabolism and nutrition disorders	5142	2.27	1489	2.51	805	4.09
Cardiac disorders	1549	0.68	1235	2.08	240	1.22
Vascular disorders	3829	1.69	1032	1.74	354	1.80
Hepatobiliary disorders	1328	0.59	964	1.62	447	2.27
Renal and urinary disorders	1991	0.88	800	1.35	359	1.82
Immune system disorders	1455	0.64	789	1.33	66	0.34
Eye disorders	2845	1.26	765	1.29	129	0.66
Reproductive system and breast disorders	811	0.36	437	0.74	40	0.20
Surgical and medical procedures	2332	1.03	161	0.27	746	3.79
Ear and labyrinth disorders	1436	0.63	151	0.25	47	0.24
Product issues	383	0.17	133	0.22	13	0.07
Endocrine disorders	196	0.09	81	0.14	11	0.06
Congenital, familial and genetic disorders	73	0.03	54	0.09	6	0.03
Social circumstances	456	0.20	20	0.03	13	0.07
Pregnancy, puerperium and perinatal conditions	13	0.01	1	0.00	1	0.01
Total	226622	100	59437	100	19672	100

*SOCs are presented in alphabetical order.

We further analyzed the venous thromboembolism events (TE), interstitial lung disease (ILD), and QT prolongation associated with CDK4/6 inhibitors. A total of 310 ILD, 3,377 VTE, and 408 QT prolongation reports were found in FAERS, as of January 2024. A disproportionality signal was found for TE and CDK4/6 inhibitors as a class and as individual drugs with Ribociclib (ROR 1.67, 95% CI 1.41-1.99), Abemaciclib (ROR 6.84, 95% CI 6.08-7.71), and Palbociclib (ROR 1.65, 95% CI 1.51-1.81). The ROR values of the “interstitial lung disease” were significant for Abemaciclib and Ribociclib (ROR 10.38, 95% CI 2.48-56.68; ROR 1.82, 95% CI 1.59-2.07). Disproportionality analyses for “Torsade de pointes/QT prolongation” showed that only Ribociclib was a positive signal(ROR 6.16, 95%CI 5.54-6.84). A summary of the key safety profiles for each CDK4/6 inhibitor, integrating findings from both the network meta-analysis and the disproportionality analysis, is provided in **Supplementary Table S4**.

#### 3.2.3 Serious adverse reactions related to CDK4/6 inhibitors

Serious adverse events were detected for all three drugs, with Ribociclib having a relatively low number of 3,397 cases (25.31%), Palbociclib with 32,745 (46.63%), and Abemaciclib with 4,042 cases (46.05%). Regarding outcomes of the detected safety signals, Ribociclib had the highest percentage of death among the studied drugs (4.76%), and Palbociclib and Abemaciclib each accounted for 3.83%. Palbociclib had the highest percentage of major events (13.22%).

#### 3.2.4 Time to onset of CDK4/6 inhibitors-associated adverse events

**Figure 6:**
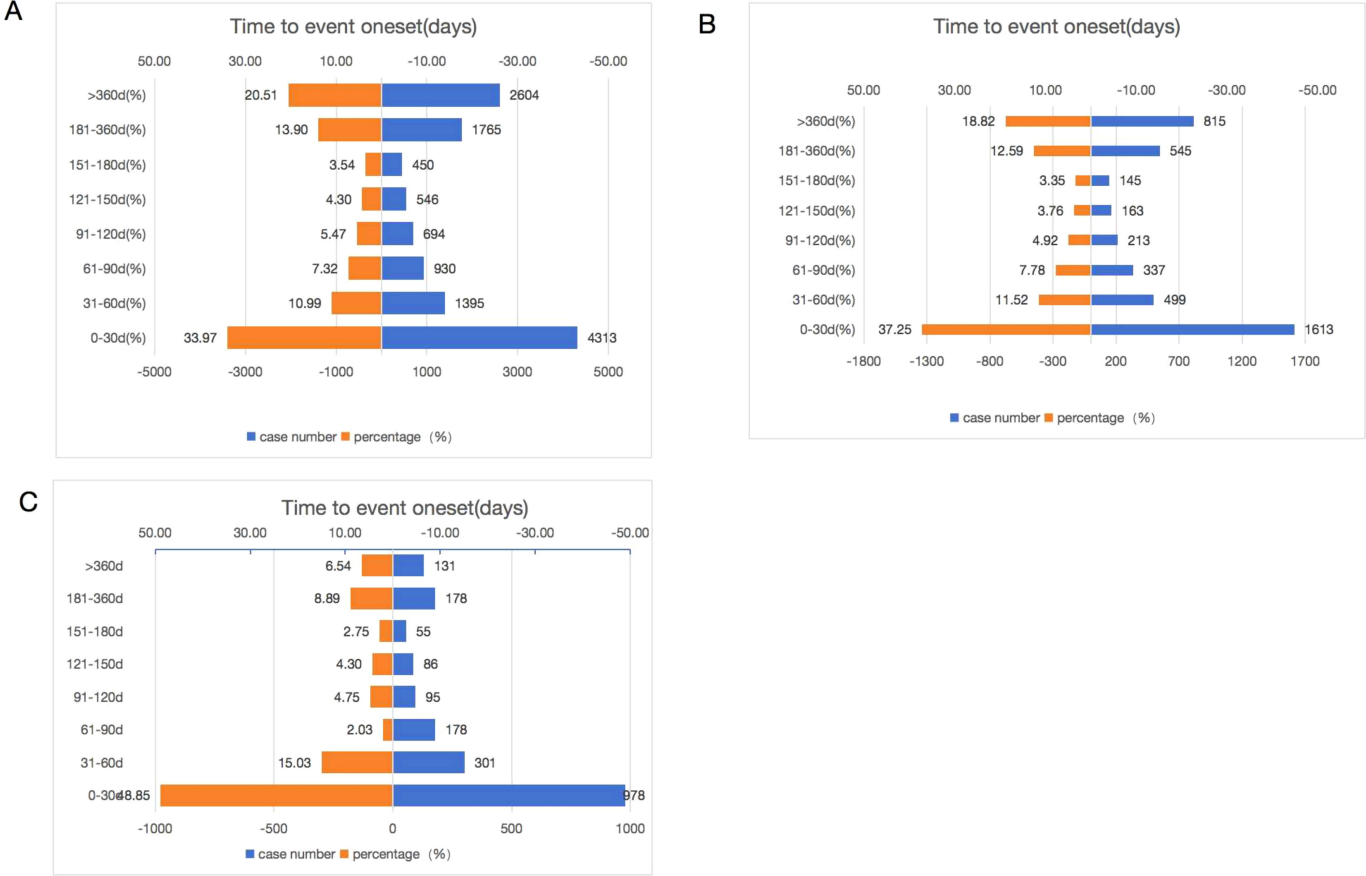
Time to onset of Palbociclib **(A)**, Ribociclib **(B)**, and Abemaciclib **(C)** associated adverse events.

Of the total adverse events reported, 12,697 were reported for Palbociclib, 2002 for Abemaciclib, and 4,330 for Ribociclib, containing comprehensive and accurate details regarding the time of occurrence. The median onset time of AEs for Ribociclib, Abemaciclib, and Palbociclib was 32 days (IQR of 11–102 days), 64 days (IQR of 16–264 days), and 81 days (IQR of 20–285 days), respectively. As shown in **Figure 6**, the majority of AEs occurred within the first month of using CDK4/6 inhibitors. AEs were least likely to occur during the second to sixth months of treatment, but significantly rose afterward, both as a drug class and as a single agent. Significantly, our data revealed that a considerable 20.51% and 18.82% of AEs may still occur following a year of treatment in Palbociclib and Ribociclib. These findings emphasize the importance of monitoring patients for potential AEs throughout the course of CDK4/6 inhibitor therapy, even beyond the initial months.

## 4 Discussion

Our integrated analysis of clinical trials and real-world pharmacovigilance data reveals distinct safety profiles among CDK4/6 inhibitors. The network meta-analysis of 16 RCTs (n=6,722) indicated that Abemaciclib or Palbociclib combinations posed a higher risk of severe adverse events, with Palbociclib conferring greater hematological risks (neutropenia, thrombocytopenia, and anemia) and Abemaciclib a greater risk of diarrhea. Disproportionality analysis of 305,731 FAERS reports further differentiated the drugs, linking Abemaciclib to gastrointestinal and metabolic disorders, and Palbociclib and Ribociclib to reports concerning neoplasms, skin disorders, and nervous system disorders.

With the widespread use of CDK4/6 inhibitors in ER-positive breast cancer, the associated AEs were significantly increased and affected several organs or tissues. No statistically significant difference in grade 1–5 adverse events was observed between the treatment combinations of the three CDK4/6 inhibitors in our network meta-analysis. However, grade 3+ hematological toxicity was mostly represented by neutropenia, with a higher incidence observed with Palbociclib and Ribociclib compared with Abemaciclib, because Abemaciclib shows greater selectivity to CDK4 (29). Some of our findings are consistent with previously published studies that compared the safety of different CDK4/6 inhibitors. Liu et al. reported that treatment combinations involving three CDK4/6 inhibitors plus AI or Fulvestrant were associated with a higher incidence of severe adverse events compared with AI or Fulvestrant alone (30). However, neutropenia induced by CDK4/6 inhibitors usually decreases with subsequent cycles. This data suggested that there is no cumulative toxicity, and the onset of neutropenia rarely led to treatment discontinuation (31).

Gastrointestinal events are frequent during CDK4/6 inhibitor treatment, including nausea, vomiting, and diarrhea, of which diarrhea is the most common event. We revealed significant disproportionality of diarrhea at specific SMQs and PT levels in these three CDK4/6 inhibitors. Network meta results showed that Abemaciclib was associated with a higher incidence and severity of diarrhea compared with Palbociclib and Ribociclib. In general, diarrhea was experienced within 1 week of Abemaciclib initiation. Despite high levels of diarrhea incidence, there was a quick resolution with a median duration of 7.5 days (Grade 2) and 4.5 days (Grade 3) (32). As for nausea and vomiting, they can be treated or prevented with antiemetic drugs. However, extra attention should be given while co-administering antiemetics with CDK4/6 inhibitors (especially with Ribociclib) due to the risk of QTc prolongation (33). In our study, a disproportionate association with QTc prolongation is detected for Ribociclib (ROR 9.69, 95% CI 8.68,10.81), which is strongly in agreement with the findings of the clinical trial (11). The results emphasize that Ribociclib should not be administered to patients who are at risk of developing QTc prolongation. Previous studies showed that most of these patients have changes during the first cycle of the combination therapy and were limited by dose interruption or reduction (10).

Since some delayed or rare AEs cannot be observed within clinical timescales, evidence from real-world data in large samples can fill this gap. To date, several disproportionality analyses have investigated AEs associated with CDK4/6 inhibitors with VTE and ILD. Raschi et al. (34) summarized 1,722 cases of TE and found disproportionate signals for VTE and CDK4/6 inhibitors as a class of drugs and as individual drugs, consistent with our findings. The previous study presented that Palbociclib and Ribociclib carried a higher risk of venous thromboembolism event (35). In a real-world setting, Abemaciclib was associated with a VTE rate approximately twofold greater than the already elevated rates reported in the MONARCH trials (36). The drug received a black box warning from the U.S. FDA about the risk of VTE. Our study confirms that this labeling is not only warranted but that thrombosis rates appear to be higher in real-world populations. Regarding pulmonary toxicity, Raschi et al. (37) reported 161 cases of ILD related to CDK4/6 inhibitors as of 2020, and Abemaciclib was found with higher-than-expected reporting, which was consistent with our study results. Their study provided the first systematic pharmacovigilance assessment of ILD by integrating FAERS data with cases from RCTs and implementing rigorous bias controls. It established a strong and consistent drug-specific signal for Abemaciclib, suggesting a risk beyond a class effect. Building upon this foundation, our updated analysis, which encompasses more recent data, confirms the persistent and significant signal for Abemaciclib. With the widespread use of CDK4/6 inhibitors, reporting of ILD was continuously increasing, and our present study found that Ribociclib was also a positive signal. When looking at the pneumotox.com (last accessed April 2, 2024), a standard reference for ILD, the statement “pneumonitis/ILD” is reported for Palbociclib and Abemaciclib, whereas a general reference to the FDA warning on CDK4/6 inhibitors as a class is mentioned for Ribociclib. Therefore, during the use of CDK4/6 inhibitors, ILD should be closely monitored, and the importance of early recognition of signs/symptoms suggestive of ILD (e.g., dyspnea) should be strengthened, especially at the beginning of treatment.

Furthermore, our analysis identified several unexpected AEs not currently highlighted in the drug labels. The signal for peripheral neuropathy associated with Palbociclib, whereas requiring further clinical confirmation, is mechanistically plausible. Although CDK4/6 inhibitors are designed to target cell cycle kinases, off-target effects on other structurally related kinases cannot be ruled out. Notably, cyclin-dependent kinase 5 (CDK5), a close family member, is abundantly expressed in post-mitotic neurons and plays a critical role in neuronal survival, axonal transport, and synaptic plasticity. Preclinical evidence has demonstrated that pharmacological inhibition of CDK5 activity can modulate pain-related neuronal plasticity and nociceptive signaling in the peripheral and central nervous systems (38). Therefore, it is conceivable that off-target inhibition of neuronal CDK5 (or other related kinases) by CDK4/6 inhibitors could disrupt normal neuronal function and contribute to the development of peripheral neuropathy in a subset of patients. Similarly, the signal for decreased blood potassium (hypokalemia) with Abemaciclib may be indirectly linked to its well-characterized propensity to cause diarrhea. Profuse diarrhea can lead to significant gastrointestinal potassium loss, resulting in hypokalemia, a well-established pathophysiological mechanism. The clinical significance of these unexpected signals warrants careful consideration. For peripheral neuropathy associated with Palbociclib, biological plausibility suggests maintaining a low threshold for clinical suspicion. Therefore, clinicians should consider asking about neuropathy symptoms during follow-up, especially in patients receiving long-term treatment. In contrast, signals such as dysphagia or speech disorders, although statistically significant, have no clear mechanistic link and can be monitored routinely. These hypotheses, while speculative, provide a rationale for heightened clinical awareness and future research into these potential toxicities.

Overall, our findings demonstrated that the risk profiles of CDK4/6 inhibitors are different; clinicians should consider specific risk factors to select the optimal treatment agent for the individual patient. However, some limitations should be recognized in the approach of the data mining procedure using the FAERS database. Firstly, as a spontaneous reporting system, FAERS is subject to well-documented biases, including significant underreporting, where it is estimated that only a small fraction of all ADRs are ever reported (39). Furthermore, reporting is influenced by factors other than a drug’s true risk profile, such as the Weber effect (higher reporting rates in the initial years post-approval), media attention, and the novelty of a drug or its adverse events. The high proportion of consumer reports in our dataset (e.g., exceeding 50% for Ribociclib) introduces a potential for reporting bias, as these reports may lack the clinical detail and diagnostic accuracy of those from healthcare professionals. This could lead to an overestimation of subjective or less severe AEs (e.g., “feeling abnormal”) and an under-ascertainment of correctly graded laboratory abnormalities. To partially overcome these fundamental limitations and provide risk estimates that are more reflective of real-world use, recent pharmacovigilance research has demonstrated the value of linking adverse event data to drug exposure data. As exemplified by Mokbel et al., who mapped the UK’s Yellow Card data onto national prescribing data to calculate reporting rates per million prescription items, this approach can create more robust comparative safety profiles and mitigate the confounding effects of differential drug usage and reporting biases (40). While our study did not have access to compatible prescription volume data for such normalization, we explicitly acknowledge this as a key limitation. The signals we detected should therefore be interpreted as measures of disproportionate reporting rather than direct incidence rates, and future studies incorporating exposure data are warranted to confirm and quantify these risks. Given these limitations, further studies are needed to confirm our findings. However, the FAERS database remains an important tool that the FDA continues to use for drug post-market surveillance.

## 5 Conclusion

CDK4/6 inhibitors are integral to the treatment of HR-positive breast cancer, yet they exhibit distinct safety profiles, as evidenced by our analysis of FAERS data. These differences in toxicity may influence treatment adherence and outcomes. To support clinical decision-making, we have developed a practical monitoring flowchart (see **Supplementary Figure S1**) that summarizes key safety signals and provides surveillance strategies for each agent, facilitating personalized patient management and optimized drug safety.

## Data Availability

The original contributions presented in the study are included in the article/**Supplementary Material**. Further inquiries can be directed to the corresponding author.
