# Supplementary material for: Toxicity profiles of cyclin-dependent kinase 4/6 inhibitors: safety analysis from clinical trials and the FDA adverse event reporting system
Source: Front Oncol. 2025 Dec 11;15:1673284. doi: 10.3389/fonc.2025.1673284 (PMC12738172; doi:10.3389/fonc.2025.1673284)
Supplement: Supplementary file 1 [file SupplementaryFile1.docx]

**Supplementary Material**

**Table S1. Search strategies**

**Pubmed n=113**

| **No.** | **Query** | **Results** |
| --- | --- | --- |
| 10 | #9; Filters: Humans, from 2022-2024.2 | 113 |
| 9 | #3 and #4 and #8 | 421 |
| 8 | #5 or #6 or #7 | 1,711,632 |
| 7 | ((placebos[Title/Abstract]) OR (placebo*[Title/Abstract])) OR (random*[Title/Abstract]) | 1,577,585 |
| 6 | (((singl*[Title/Abstract]) OR (doubl*[Title/Abstract])) OR (trebl*[Title/Abstract])) OR (tripl*[Title/Abstract]) AND ((mask*[Title/Abstract]) OR (blind*[Title/Abstract])) | 231589 |
| 5 | clinical trial[Title/Abstract] | 210376 |
| 4 | (((((cyclin-dependent kinase 4[Title/Abstract] AND 6 inhibitor*[Title/Abstract]) OR (cyclin-dependent kinase 4/6 inhibitor*[Title/Abstract])) OR (CDK4[Title/Abstract] AND CDK6 inhibitor*[Title/Abstract])) OR (CDK4/6 inhibitor*[Title/Abstract])) OR (CDK inhibitor*[Title/Abstract])) OR (CDKi[Title/Abstract]) OR ((palbociclib[Title/Abstract]) OR (ribociclib[Title/Abstract])) OR (abemaciclib[Title/Abstract]) | 8119 |
| 3 | #1 and #2 | 107178 |
| 2 | (((metastatic[Title/Abstract]) OR (advanced[Title/Abstract])) OR (metastases[Title/Abstract])) OR (metastasis[Title/Abstract]) | 1123505 |
| 1 | "Breast Neoplasms"[Mesh] OR (((((((Breast Neoplasm*[Title/Abstract]) OR (Breast Tumor*[Title/Abstract])) OR (Breast Cancer*[Title/Abstract])) OR (Breast Carcinoma*[Title/Abstract])) OR (Mammary Neoplasm*[Title/Abstract])) OR (Mammary Tumor*[Title/Abstract])) OR (Mammary Cancer*[Title/Abstract])) OR (Mammary Carcinoma*[Title/Abstract]) | 478080 |

**Embase n=252**

| **No.** | **Query** | **Results** |
| --- | --- | --- |
| #22 | #5 AND #8 AND #20 AND ([controlled clinical trial]/lim OR [randomized controlled trial]/lim) AND [humans]/lim AND [clinical study]/lim AND [embase]/lim AND [2022-2024]/py | 252 |
| #21 | #5 AND #8 AND #20 | 1096 |
| #20 | #13 OR #14 OR #15 OR #16 OR #17 OR #18 OR #19 | 432535 |
| #19 | 'clinical trial'/exp AND ([controlled clinical trial]/lim OR [randomized controlled trial]/lim) | 117837 |
| #18 | 'single blind procedure'/exp | 8395 |
| #17 | 'double blind procedure'/exp | 23178 |
| #16 | 'randomization'/exp | 5942 |
| #15 | 'controlled clinical trial'/exp | 118610 |
| #14 | 'randomized controlled trial'/exp | 114018 |
| #13 | #11 OR #12 | 432461 |
| #12 | clinical trial OR placebos OR placebo* OR random* | 424976 |
| #11 | #9 AND #10 | 43669 |
| #10 | mask* OR blind* | 87711 |
| #9 | singl* OR doubl* OR trebl* OR tripl* | 518549 |
| #8 | #6 OR #7 | 11136 |
| #7 | palbociclib OR ribociclib OR abemaciclib | 3504 |
| #6 | (((('cyclin dependent' AND kinase AND 4 AND 6 AND inhibitor* OR (cdk4 AND cdk6 AND inhibitor*) OR 'cyclin dependent') AND kinase AND 4 AND 6 AND inhibitor* OR cdk4) AND 6 AND inhibitor* OR cdk) AND inhibitor* OR cdki) | 9365 |
| #5 | #3 AND #4 | 26230 |
| #4 | (metastatic OR advanced OR 'metastases'/exp OR 'metastasis'/exp) | 337443 |
| #3 | #1 OR #2 | 81194 |
| #2 | 'breast tumor'/exp | 80645 |
| #1 | (((((((breast AND neoplasm* OR breast) AND tumor* OR breast) AND cancer* OR breast) AND carcinoma* OR mammary) AND neoplasm* OR mammary) AND tumor* OR mammary) AND cancer* OR mammary) AND carcinoma* | 4240 |

**ClinicalTrials.gov n=53**

| 1 | Patient | breast tumor |
| --- | --- | --- |
| 2 | Intervention | “palbociclib” OR “ribociclib” OR “abemaciclib” |
| 3 | Study phase | Phase II and phase III clinical trial |
| 4 | Study status | Completed and with results |

**Table S2.Baseline characteristics of 16 studies**

| **Study** | **Clinicaltrial ID** | **Author** | **Year** | **Phase** | **Line** | **Sample size** | **Median age, years** | **Arm** | **Treatment** |
| --- | --- | --- | --- | --- | --- | --- | --- | --- | --- |
| PALOMA-1 | NCT00721409 | Finn RS | 2020 | II | First-line | 165  (84 vs 81) | 63 vs64 | 1 | Palbociclib 125mg daily, 3 weeks on /1 week off;letrozole 2.5mg qd |
|  |  |  |  |  |  |  |  | 2 | letrozole 2.5mg qd |
| PALOMA-2 | NCT01740427 | Rugo HS | 2019 | II | First-line | 666  (444 vs 222) | 62 vs 61 | 1 | Palbociclib 125mg daily, 3 weeks on/1 week off;letrozole 2.5mg qd |
|  |  |  |  |  |  |  |  | 2 | letrozole 2.5mg qd |
| PALOMA-3 | NCT01942135 | Turner NC | 2018 | III | Second- and subsequent line | 521  (347 vs 174) | 57 vs 56 | 1 | Palbociclib 125mg daily 3 weeks on/1 week off;fulvestrant 500mg q4w |
|  |  |  |  |  |  |  |  | 2 | fulvestrant 500mg q4w |
| MONALEESA-2 | NCT01958021 | Hortobagyi GN | 2018 | III | First-line | 668  (334 vs 334) | 62 vs 63 | 1 | Ribociclib 600mg daily 3 weeks on/1 week off;letrozole 2.5mg qd |
|  |  |  |  |  |  |  |  | 2 | letrozole 2.5mg qd |
| MONALEESA-3 | NCT02422615 | Slamon DJ | 2021 | III | First-line | 726  (484 vs 242) | 63 vs 63 | 1 | Ribociclib 600mg daily 3 weeks on/ 1 week off;fulvestrant 500mg q4w |
|  |  |  |  |  |  |  |  | 2 | fulvestrant 500mg q4w |
| MONALEESA-7 | NCT02278120 | Im SA | 2019 | III | First-line | 672  (335 vs 337) | 43 vs 45 | 1 | Ribociclib 600mg daily 3 weeks on/1 week off; 20mg qd; tamoxifen 20mg qd or letrozole 2.5mg qd or anastrozole 1mg qd; goserelin 3.6mg q4w |
|  |  |  |  |  |  |  |  | 2 | tamoxifen 20mg qd or letrozole 2.5mg qd or anastrozole 1mg qd; goserelin 3.6mg q4w |
| MONARCH-2 | NCT02107703 | Sledge GW Jr | 2020 | III | Second-line | 669  (446 vs 223) | 59 vs 62 | 1 | Abemaciclib 150mg bid; fulvestrant 500mg q4w |
|  |  |  |  |  |  |  |  | 2 | fulvestrant 500mg q4w |
| MONARCH-3 | NCT02246621 | Johnston S | 2019 | III | First-line | 493  (328 vs 165) | 63 vs 63 | 1 | Abemaciclib 150mg bid; letrozole 2.5mg qd or anastrozole 1mg qd. |
|  |  |  |  |  |  |  |  | 2 | letrozole 2.5mg qd or anastrozole 1mg qd. |
| MONARCH plus | NCT02763566 | Zhang QY | 2020 | III | First-line | 306  (207 vs 99) | n.r. | 1 | Abemaciclib 150mg bid; letrozole 2.5mg qd or anastrozole 1mg qd |
| (cohort A) |  |  |  |  |  |  |  | 2 | letrozole 2.5mg qd or anastrozole 1mg qd |
| MONARCH plus | NCT02763566 | Zhang QY | 2020 | III | First- and subsequent-line | 157  (104 vs 53) | n.r. | 1 | Abemaciclib 150mg bid; fulvestrant 500mg q4w |
| (cohort B) |  |  |  |  |  |  |  | 2 | fulvestrant 500mg q4w |
| PALOMA-4 | NCT02297438 | Xu BH | 2022 | III | First-line | 340（169vs171） | 54vs54 | 1 | palbociclib (125 mg/d orally; 3 weeks on, 1 week off) plus letrozole (2.5 mg/d orally; continuously) |
|  |  |  |  |  |  |  |  | 2 | placebo plus letrozole |
| nextMONARCH | NCT02747004 | Hamilton E | 2022 | Ⅱ | Second-line | 157  （78vs79） | n.r. | 1 | abemaciclib (150 mg BID) with tamoxifen (20 mg daily) (A +T) |
|  |  |  |  |  |  |  |  | 2 | abemaciclib monotherapy (150 mg BID) |
| AMICA | NCT03555877 | Decker T | 2023 | Ⅱ | Second- and subsequent line | 53  （43vs10) | 61vs70 | 1 | ribociclib (starting at 600 mg daily, 3 weeks on and 1 week off) ;standard ET (tamoxifen, anastrozole, letrozole, exemestane, or fulvestrant; additional luteinizing hormone releasing hormone (LHRH) analogue for premenopausal women) as maintenance therapy. |
|  |  |  |  |  |  |  |  | 2 | standard ET (tamoxifen, anastrozole, letrozole, exemestane, or fulvestrant; additional luteinizing hormone releasing hormone (LHRH) analogue for premenopausal women) as maintenance therapy. |
| PEARL | NCT02028507 | Martı´n M | 2022 | III | First- and subsequent-line | 299（149vs150） | 60vs62 | 1 | palbociclib plus fulvestrant (500 mg on days 1 and 15 of cycle 1 and then on day 1 every four weeks) |
|  |  |  |  |  |  |  |  | 2 | palbociclib plus fulvestrant (500 mg on days 1 and 15 of cycle 1 and then on day 1 every four weeks) |
| FLIPPER | NCT02690480 | Albanell.J | 2021 | Ⅱ | First- and subsequent-line | 189  （94vs95） | 64vs64 | 1 | palbociclib (125 mg/day, 28-day cycles; 3weeks on, 1 week off) ；fulvestrant (500 mg on days 1, 14, 28, andevery 28 days onward). |
|  |  |  |  |  |  |  |  | 2 | fulvestrant (500 mg on days 1, 14, 28, and every 28 days onward). |
| PARSIFAL | NCT02491983 | Cosimo S D | 2022 | III | Second- and subsequent line | 483（241vs242) | 64vs62 | 1 | palbociclib at 125 mg per day (3 weeks on, 1 week off) plus; intramuscular fulvestrant at 500 mg on days 1, 15, 29, and once monthly thereafter） |
|  |  |  |  |  |  |  |  | 2 | palbociclib at 125 mg per day (3 weeks on, 1 week off) plus letrozole at 2.5 mg per day (continuous treatment) |
| monarcHER | NCT02675231 | Tolaney S M | 2024 | Ⅱ | Second- and subsequent line | 158  (79vs79) | n.r. | 1 | Abemaciclib 150 mg bid.Trastuzumab 8 mg/kg on Day 1 of Cycle 1, then maintained at 6 mg/kg on Day 1 of each subsequent 21-day cycle;Fulvestrant 500 mg on Days 1 and 15 of Cycle 1, and on Day 29 (Day 8 of Cycle 2 if no dose suspension for trastuzumab occurred), then once every 4 weeks |
|  |  |  |  |  |  |  |  | 2 | Abemaciclib 150 mg bid.Trastuzumab 8 mg/kg on Day 1 of Cycle 1, then maintained at 6 mg/kg on Day 1 of each subsequent 21-day cycle |

**Table S3. Characteristics of reports associated with CDK4/6 inhibitors from January 2011 to June 2024.**

|  | Palbociclib | Ribociclib | Abemaciclib |
| --- | --- | --- | --- |
| Number of events | 70227 | 13422 | 8778 |
| Age distribution, n (%) | | | |
| ＜18y | 63( 0.09) | 19( 0.14) | 4( 0.05) |
| ≥18y，＜45y | 3325( 4.73) | 862( 6.42) | 292( 3.33) |
| ≥45y，＜65y | 23784(33.87) | 2844(21.19) | 1786(20.35) |
| ≥65y，＜75y | 18989(27.04) | 1594(11.88) | 1192(13.58) |
| ≥75y | 14912(21.23) | 974( 7.26) | 780( 8.89) |
| Missing | 9154(13.03) | 7129(53.11) | 4724(53.82) |
| Sex, n (%) | | | |
| Female | 65761(93.64) | 12244(91.22) | 7808(88.95) |
| Male | 1613( 2.30) | 244( 1.82) | 152( 1.73) |
| Missing | 2853( 4.06) | 934( 6.96) | 818( 9.32) |
| Report source, n (%) | | | |
| **Physician** | **9394(13.38)** | **3391(25.26)** | **1180(13.44)** |
| **Pharmacist** | **20521(29.22)** | **1989(14.82)** | **2027(23.09)** |
| **Consumer** | **30408(43.30)** | **7038(52.44)** | **4262(48.55)** |
| **Other** | **9904(14.1)** | **1004(7.48)** | **1309(14.92)** |
| **Reporter country, n (%)** |  |  |  |
| **USA** | **57558(81.96)** | **4639 (34.57)** | **6879(78.3)** |
| **Other countries** | **12669(18.04)** | **8783(65.43)** | **1899(21.7)** |

**Table S4 1-3**

**Table S4-1. Signal strength of reports of Abemaciclib at the PT level in the FAERS database.**

| SOC Name | Perferred terms (PTs) | **Case Numbers** | **ROR** | **95%CI Lower** | **95%CI Upper** |
| --- | --- | --- | --- | --- | --- |
| Blood and lymphatic system disorders | Anaemia | 198 | 3.229 | 2.807 | 3.714 |
| Blood and lymphatic system disorders | Neutropenia | 198 | 4.795 | 4.168 | 5.516 |
| Blood and lymphatic system disorders | Myelosuppression | 146 | 23.699 | 20.121 | 27.914 |
| Blood and lymphatic system disorders | Thrombocytopenia | 73 | 2.192 | 1.742 | 2.759 |
| Vascular disorders | Thrombosis | 75 | 2.940 | 2.343 | 3.689 |
| Vascular disorders | Hot flush* | 48 | 2.099 | 1.581 | 2.786 |
| Vascular disorders | Deep vein thrombosis | 47 | 2.200 | 1.652 | 2.929 |
| Gastrointestinal disorders | Diarrhoea | 2420 | 13.287 | 12.733 | 13.866 |
| Gastrointestinal disorders | Nausea | 690 | 2.864 | 2.654 | 3.090 |
| Gastrointestinal disorders | Vomiting | 456 | 3.236 | 2.949 | 3.551 |
| Gastrointestinal disorders | Abdominal pain upper* | 179 | 2.755 | 2.377 | 3.192 |
| Gastrointestinal disorders | Abdominal pain* | 165 | 2.277 | 1.953 | 2.654 |
| Gastrointestinal disorders | Constipation* | 146 | 2.200 | 1.869 | 2.589 |
| Gastrointestinal disorders | Abdominal discomfort | 95 | 1.696 | 1.386 | 2.074 |
| Gastrointestinal disorders | Gastrointestinal disorder | 65 | 1.690 | 1.325 | 2.157 |
| Gastrointestinal disorders | Stomatitis | 60 | 3.110 | 2.413 | 4.007 |
| Gastrointestinal disorders | Dysphagia* | 49 | 1.588 | 1.199 | 2.101 |
| Renal and urinary disorders | Acute kidney injury* | 99 | 1.555 | 1.277 | 1.895 |
| Renal and urinary disorders | Renal impairment* | 67 | 2.496 | 1.964 | 3.173 |
| General disorders and administration site conditions | Fatigue | 660 | 2.630 | 2.434 | 2.843 |
| General disorders and administration site conditions | Drug ineffective | 495 | 1.116 | 1.021 | 1.220 |
| General disorders and administration site conditions | Death | 485 | 1.694 | 1.548 | 1.853 |
| General disorders and administration site conditions | Disease progression | 234 | 6.478 | 5.694 | 7.371 |
| General disorders and administration site conditions | Asthenia | 227 | 1.922 | 1.687 | 2.191 |
| General disorders and administration site conditions | Drug intolerance | 104 | 3.296 | 2.718 | 3.997 |
| General disorders and administration site conditions | Illness | 64 | 2.679 | 2.096 | 3.424 |
| Skin and subcutaneous tissue disorders | Alopecia* | 183 | 2.692 | 2.327 | 3.114 |
| Respiratory, thoracic and mediastinal disorders | Interstitial lung disease | 136 | 9.565 | 8.077 | 11.326 |
| Respiratory, thoracic and mediastinal disorders | Pneumonitis | 95 | 11.790 | 9.633 | 14.431 |
| Respiratory, thoracic and mediastinal disorders | Pulmonary embolism | 94 | 3.133 | 2.558 | 3.837 |
| Investigations | White blood cell count decreased | 274 | 7.939 | 7.045 | 8.947 |
| Investigations | Weight decreased* | 184 | 2.107 | 1.822 | 2.436 |
| Investigations | Blood creatinine increased* | 170 | 8.896 | 7.647 | 10.349 |
| Investigations | Platelet count decreased | 134 | 4.001 | 3.376 | 4.743 |
| Investigations | Haemoglobin decreased | 101 | 3.164 | 2.601 | 3.847 |
| Investigations | Red blood cell count decreased | 77 | 8.484 | 6.780 | 10.617 |
| Investigations | Neutrophil count decreased | 73 | 5.913 | 4.697 | 7.442 |
| Investigations | Full blood count decreased | 61 | 8.728 | 6.785 | 11.227 |
| Investigations | Full blood count abnormal | 46 | 4.809 | 3.600 | 6.425 |
| Hepatobiliary disorders | Hepatic function abnormal | 84 | 8.121 | 6.552 | 10.066 |
| Hepatobiliary disorders | Hepatotoxicity | 50 | 7.556 | 5.722 | 9.978 |
| Hepatobiliary disorders | Liver disorder | 48 | 3.715 | 2.798 | 4.932 |
| Metabolism and nutrition disorders | Decreased appetite | 323 | 4.352 | 3.898 | 4.858 |
| Metabolism and nutrition disorders | Dehydration* | 280 | 6.912 | 6.142 | 7.779 |
| Investigations | Alanine aminotransferase increased | 42 | 2.592 | 1.914 | 3.509 |
| Investigations | Hepatic enzyme increased | 41 | 2.092 | 1.540 | 2.843 |
| Investigations | Aspartate aminotransferase increased | 40 | 2.971 | 2.178 | 4.053 |
| Hepatobiliary disorders | Drug-induced liver injury | 40 | 4.287 | 3.143 | 5.848 |
| Investigations | Liver function test increased | 38 | 5.596 | 4.069 | 7.696 |
| Investigations | Blood potassium decreased* | 37 | 4.000 | 2.896 | 5.524 |

Notes: *, AEs that are not mentioned in the drug label. PT, Preferred Terms.

**Table S4-2. Signal strength of reports of Palbociclib at the PT level in the FAERS database**

| **SOC Name** | **Perferred terms (PTs)** | **Case Numbers** | **ROR** | **95%CI Lower** | **95%CI Upper** |
| --- | --- | --- | --- | --- | --- |
| Blood and lymphatic system disorders | Neutropenia | 3715 | 8.133 | 7.869 | 8.407 |
| Blood and lymphatic system disorders | Anaemia | 1345 | 1.903 | 1.803 | 2.008 |
| Blood and lymphatic system disorders | Bone marrow failure | 896 | 11.499 | 10.748 | 12.302 |
| Blood and lymphatic system disorders | Leukopenia | 817 | 4.850 | 4.524 | 5.199 |
| Blood and lymphatic system disorders | Thrombocytopenia | 661 | 1.727 | 1.600 | 1.865 |
| Vascular disorders | Hot flush* | 1400 | 5.454 | 5.171 | 5.753 |
| Gastrointestinal disorders | Nausea | 6421 | 2.311 | 2.254 | 2.369 |
| Gastrointestinal disorders | Diarrhoea | 4359 | 1.857 | 1.802 | 1.914 |
| Gastrointestinal disorders | Vomiting | 2379 | 1.449 | 1.391 | 1.509 |
| Gastrointestinal disorders | Stomatitis | 2253 | 10.708 | 10.261 | 11.174 |
| Gastrointestinal disorders | Constipation* | 2116 | 2.797 | 2.679 | 2.921 |
| Gastrointestinal disorders | Abdominal discomfort | 1066 | 1.657 | 1.559 | 1.760 |
| Gastrointestinal disorders | Oral pain | 643 | 7.759 | 7.170 | 8.396 |
| Gastrointestinal disorders | Dyspepsia | 618 | 1.757 | 1.623 | 1.902 |
| Gastrointestinal disorders | Dysphagia* | 607 | 1.713 | 1.582 | 1.856 |
| General disorders and administration site conditions | Fatigue | 12035 | 4.317 | 4.238 | 4.398 |
| General disorders and administration site conditions | Malaise | 2749 | 1.576 | 1.518 | 1.637 |
| General disorders and administration site conditions | Asthenia | 2676 | 1.977 | 1.903 | 2.054 |
| General disorders and administration site conditions | Pain* | 2585 | 1.047 | 1.007 | 1.089 |
| General disorders and administration site conditions | Feeling abnormal* | 1017 | 1.086 | 1.021 | 1.155 |
| General disorders and administration site conditions | Peripheral swelling* | 875 | 1.401 | 1.311 | 1.498 |
| Skin and subcutaneous tissue disorders | Alopecia | 5125 | 6.827 | 6.637 | 7.022 |
| Skin and subcutaneous tissue disorders | Rash | 1783 | 1.070 | 1.021 | 1.121 |
| Skin and subcutaneous tissue disorders | Dry skin | 1118 | 2.278 | 2.148 | 2.417 |
| Psychiatric disorders | Insomnia* | 1126 | 1.155 | 1.089 | 1.224 |
| Respiratory, thoracic and mediastinal disorders | Dyspnoea | 2352 | 1.130 | 1.085 | 1.177 |
| Respiratory, thoracic and mediastinal disorders | Cough | 1711 | 1.655 | 1.578 | 1.736 |
| Respiratory, thoracic and mediastinal disorders | Epistaxis | 1062 | 3.780 | 3.557 | 4.018 |
| Respiratory, thoracic and mediastinal disorders | Oropharyngeal pain* | 555 | 1.521 | 1.399 | 1.653 |
| Musculoskeletal and connective tissue disorders | Arthralgia* | 2094 | 1.373 | 1.315 | 1.434 |
| Musculoskeletal and connective tissue disorders | Back pain* | 1362 | 1.549 | 1.468 | 1.634 |
| Musculoskeletal and connective tissue disorders | Pain in extremity* | 1306 | 1.141 | 1.080 | 1.205 |
| Musculoskeletal and connective tissue disorders | Bone pain8 | 904 | 4.146 | 3.882 | 4.429 |
| Nervous system disorders | Dizziness | 1892 | 1.052 | 1.005 | 1.101 |
| Nervous system disorders | Memory impairment | 1071 | 1.991 | 1.874 | 2.115 |
| Nervous system disorders | Neuropathy peripheral* | 982 | 2.860 | 2.685 | 3.046 |
| Nervous system disorders | Hypoaesthesia | 758 | 1.368 | 1.273 | 1.469 |
| Investigations | White blood cell count decreased | 9395 | 27.442 | 26.845 | 28.052 |
| Investigations | Full blood count abnormal | 2087 | 21.006 | 20.074 | 21.981 |
| Investigations | Platelet count decreased | 1774 | 4.685 | 4.468 | 4.911 |
| Investigations | Neutrophil count decreased | 1691 | 12.624 | 12.016 | 13.263 |
| Investigations | Full blood count decreased | 1531 | 20.988 | 19.906 | 22.129 |
| Investigations | Weight decreased | 1447 | 1.436 | 1.364 | 1.513 |
| Investigations | Red blood cell count decreased | 1406 | 14.364 | 13.604 | 15.166 |
| Investigations | Haemoglobin decreased | 1246 | 3.429 | 3.241 | 3.627 |
| Infections and infestations | Nasopharyngitis | 1090 | 1.560 | 1.470 | 1.656 |
| Infections and infestations | Urinary tract infection | 896 | 1.409 | 1.319 | 1.505 |
| Infections and infestations | Infection | 615 | 1.168 | 1.079 | 1.265 |
| Ear and labyrinth disorders | Hypoacusis* | 765 | 4.041 | 3.761 | 4.341 |
| Metabolism and nutrition disorders | Decreased appetite | 2691 | 3.163 | 3.044 | 3.287 |
| Metabolism and nutrition disorders | Dehydration | 678 | 1.436 | 1.331 | 1.549 |

Notes: *, AEs that are not mentioned in the drug label. PT, Preferred Terms.

**Table S4-3. Signal strength of reports of Ribociclib at the PT level in the FAERS database**

| **SOC Name** | **Perferred terms (PTs)** | **Case Numbers** | **ROR** | **95%CI Lower** | **95%CI Upper** |
| --- | --- | --- | --- | --- | --- |
| Blood and lymphatic system disorders | Neutropenia | 979 | 7.958 | 7.468 | 8.479 |
| Blood and lymphatic system disorders | Anaemia | 397 | 2.136 | 1.935 | 2.358 |
| Blood and lymphatic system disorders | Leukopenia | 259 | 5.789 | 5.121 | 6.544 |
| Blood and lymphatic system disorders | Thrombocytopenia | 176 | 1.749 | 1.508 | 2.028 |
| Blood and lymphatic system disorders | Pancytopenia | 97 | 1.949 | 1.597 | 2.379 |
| Cardiac disorders | Atrial fibrillation* | 127 | 1.373 | 1.154 | 1.635 |
| Eye disorders | Visual impairment | 147 | 1.181 | 1.004 | 1.389 |
| Gastrointestinal disorders | Nausea | 1537 | 2.093 | 1.989 | 2.202 |
| Gastrointestinal disorders | Vomiting | 930 | 2.169 | 2.033 | 2.314 |
| Gastrointestinal disorders | Diarrhoea | 814 | 1.309 | 1.222 | 1.403 |
| Gastrointestinal disorders | Constipation | 458 | 2.287 | 2.086 | 2.508 |
| Gastrointestinal disorders | Abdominal discomfort | 281 | 1.661 | 1.477 | 1.868 |
| Gastrointestinal disorders | Abdominal pain upper | 242 | 1.226 | 1.080 | 1.391 |
| Gastrointestinal disorders | Abdominal distension | 119 | 1.221 | 1.019 | 1.461 |
| Gastrointestinal disorders | Stomatitis | 102 | 1.747 | 1.439 | 2.122 |
| Gastrointestinal disorders | Gastritis | 83 | 3.407 | 2.746 | 4.227 |
| General disorders and administration site conditions | Fatigue | 1537 | 2.013 | 1.913 | 2.117 |
| General disorders and administration site conditions | Pain | 740 | 1.144 | 1.064 | 1.230 |
| General disorders and administration site conditions | Malaise | 704 | 1.535 | 1.425 | 1.654 |
| General disorders and administration site conditions | Asthenia | 633 | 1.774 | 1.640 | 1.918 |
| General disorders and administration site conditions | Pyrexia | 430 | 1.324 | 1.204 | 1.456 |
| General disorders and administration site conditions | Peripheral swelling* | 283 | 1.728 | 1.537 | 1.942 |
| Hepatobiliary disorders | Hepatotoxicity | 103 | 5.162 | 4.252 | 6.267 |
| Investigations | Electrocardiogram QT prolonged | 327 | 9.690 | 8.686 | 10.810 |
| Investigations | Weight decreased* | 325 | 1.226 | 1.100 | 1.368 |
| Investigations | Alanine aminotransferase increased | 219 | 4.498 | 3.937 | 5.138 |
| Investigations | Hepatic enzyme increased | 211 | 3.580 | 3.126 | 4.099 |
| Investigations | Aspartate aminotransferase increased | 204 | 5.046 | 4.396 | 5.792 |
| Investigations | Blood creatinine increased | 181 | 3.115 | 2.691 | 3.606 |
| Investigations | Blood bilirubin increased | 84 | 3.740 | 3.018 | 4.635 |
| Investigations | Blood alkaline phosphatase increased | 82 | 4.288 | 3.451 | 5.328 |
| Investigations | Transaminases increased | 82 | 3.890 | 3.131 | 4.834 |
| Metabolism and nutrition disorders | Decreased appetite | 544 | 2.409 | 2.214 | 2.622 |
| Metabolism and nutrition disorders | Dehydration* | 158 | 1.273 | 1.089 | 1.488 |
| Metabolism and nutrition disorders | Diabetes mellitus* | 93 | 1.369 | 1.117 | 1.678 |
| Musculoskeletal and connective tissue disorders | Pain in extremity* | 417 | 1.391 | 1.263 | 1.531 |
| Musculoskeletal and connective tissue disorders | Back pain | 414 | 1.794 | 1.628 | 1.976 |
| Musculoskeletal and connective tissue disorders | Spinal pain | 96 | 7.396 | 6.048 | 9.044 |
| Nervous system disorders | Speech disorder* | 80 | 1.603 | 1.287 | 1.997 |
| Nervous system disorders | Taste disorder* | 74 | 4.411 | 3.509 | 5.544 |
| Renal and urinary disorders | Renal impairment | 160 | 1.973 | 1.689 | 2.304 |
| Respiratory, thoracic and mediastinal disorders | Dyspnoea | 665 | 1.219 | 1.129 | 1.316 |
| Respiratory, thoracic and mediastinal disorders | Cough | 505 | 1.861 | 1.704 | 2.031 |
| Respiratory, thoracic and mediastinal disorders | Epistaxis* | 94 | 1.254 | 1.024 | 1.536 |
| Skin and subcutaneous tissue disorders | Alopecia | 562 | 2.741 | 2.522 | 2.979 |
| Skin and subcutaneous tissue disorders | Rash | 560 | 1.283 | 1.180 | 1.394 |
| Skin and subcutaneous tissue disorders | Pruritus | 521 | 1.457 | 1.337 | 1.589 |
| Skin and subcutaneous tissue disorders | Dry skin | 252 | 1.946 | 1.719 | 2.203 |
| Skin and subcutaneous tissue disorders | Blister | 72 | 1.372 | 1.089 | 1.729 |
| Vascular disorders | Hot flush | 160 | 2.319 | 1.985 | 2.709 |

Notes: *, AEs that are not mentioned in the drug label. PT, Preferred Terms.
