# Supplementary figures and images for: Toxicity profiles of cyclin-dependent kinase 4/6 inhibitors: safety analysis from clinical trials and the FDA adverse event reporting system
Source: Front Oncol. 2025 Dec 11;15:1673284. doi: 10.3389/fonc.2025.1673284 (PMC12738172; doi:10.3389/fonc.2025.1673284)

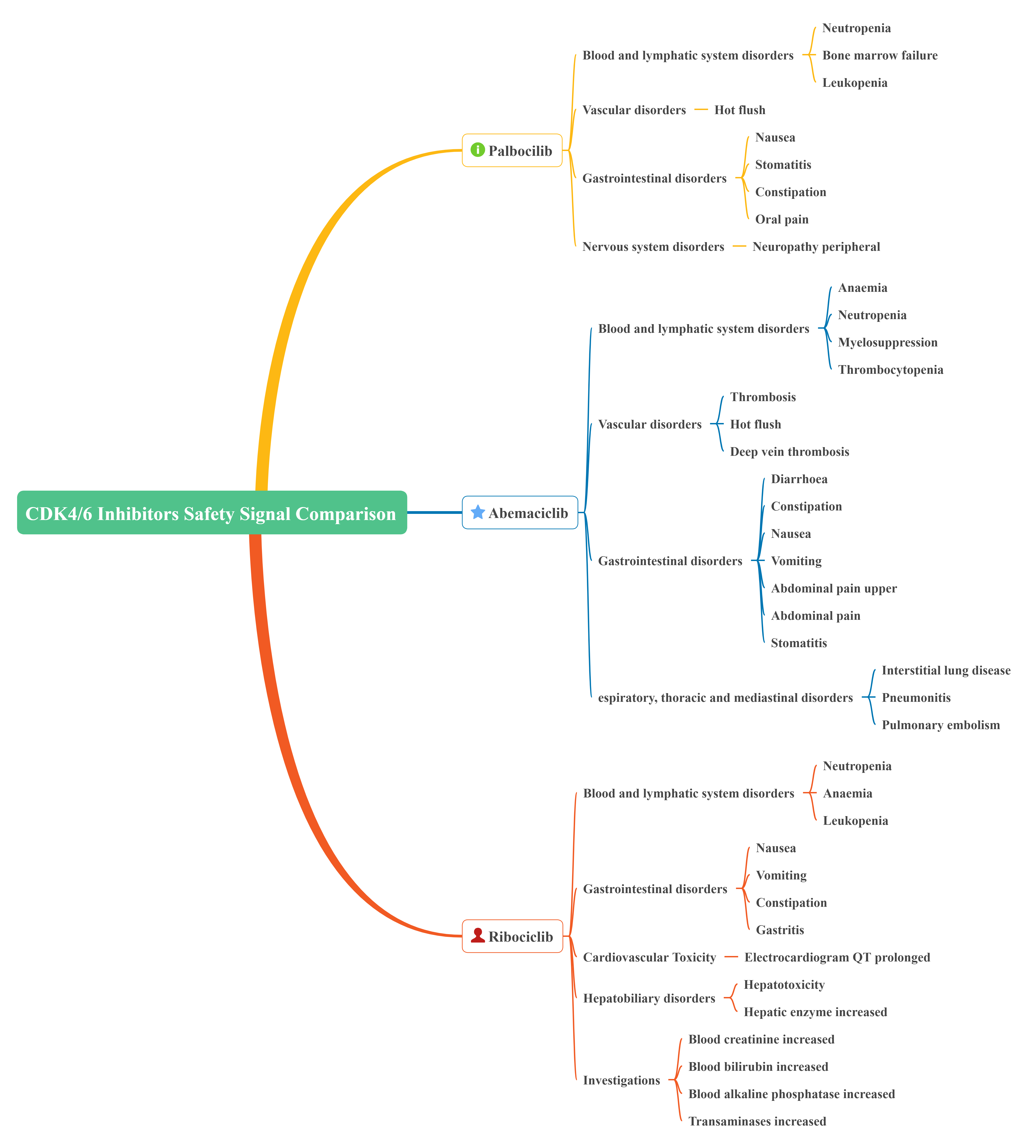

Supplement: Supplementary file 2 [file Image1.jpeg]
